# Symptoms of Obstructive Sleep Apnea, Gastroesophageal Reflux and the Risk of Barrett’s Esophagus in a Population-Based Case-Control Study

**DOI:** 10.1371/journal.pone.0129836

**Published:** 2015-06-19

**Authors:** Anna Lindam, Bradley J. Kendall, Aaron P. Thrift, Graeme A. Macdonald, Suzanne O’Brien, Jesper Lagergren, David C. Whiteman

**Affiliations:** 1 Upper GI Surgery, Department of Molecular Medicine and Surgery, Karolinska Institutet, Stockholm, Sweden; 2 Cancer Control Laboratory, QIMR Berghofer Medical Research Institute, Brisbane, Queensland, Australia; 3 Department of Medicine, School of Medicine, The University of Queensland, Brisbane, Queensland, Australia; 4 Division of Cancer Studies, King’s College London, London, United Kingdom; Hospital General Dr. Manuel Gea González, MEXICO

## Abstract

**Background:**

Gastroesophageal reflux is overrepresented in people with obstructive sleep apnea (OSA) and it has been suggested that OSA worsens gastroesophageal reflux symptoms. Aggravated reflux might lead to an increased risk of Barrett’s esophagus.

**Aim:**

To assess the association between sleep apnea symptoms and Barrett’s esophagus.

**Methods:**

Included in a case-control study in Brisbane, Australia were 237 patients with histologically confirmed Barrett’s esophagus and 247 population controls. The controls were randomly selected from the electoral roll and frequency-matched to the cases by age and sex. Information on OSA symptoms (excessive daytime sleepiness and sleep related apnea symptoms), gastroesophageal reflux symptoms and anthropometric measures were collected through interviews and written questionnaires. Multivariable logistic regression provided odds ratios (OR) and 95% confidence intervals (CI), adjusted for potential confounding by BMI and gastroesophageal reflux.

**Results:**

The prevalence of Barrett’s esophagus was higher among people with excessive daytime sleepiness than those without (24% vs. 18%; p-value 0.1142) and in participants with sleep-related apnea symptoms (20% vs. 13%; p-value 0.1730). However, there were non-significantly increased ORs of Barrett’s esophagus among people with excessive daytime sleepiness (OR 1.42, 95% CI 0.90–2.34) and sleep related apnea symptoms (OR 1.32, 95% CI 0.74–2.36) when adjusting for age, sex and BMI. After further adjustment for gastroesophageal reflux symptoms, the point ORs were no longer increased (OR 1.02, 95% CI 0.61–1.70 for daytime sleepiness and OR 0.72, 95% CI 0.38–1.38 for sleep related apnea symptoms).

**Conclusions:**

Symptoms of OSA are possibly associated with an increased risk of Barrett’s esophagus, an association that appears to be mediated entirely by gastroesophageal reflux.

## Introduction

Barrett’s esophagus, a metaplasia in the mucosal lining of the esophagus, is a precursor to esophageal adenocarcinoma. The occurrence of both these conditions has increased rapidly in Western countries, including Australia, during the last few decades [1–4]. Gastroesophageal reflux disease and obesity (abdominal adiposity in particular) are the two strongest risk factors for Barrett’s esophagus [5–7].

Obstructive sleep apnea (OSA) is characterized by recurrent airway collapse during sleep, resulting in periods of apnea and hypoapnea. OSA is defined by ≥15 apnea-hypoapnea events per hour during an overnight polysomnogram (with or without OSA symptoms) or ≥5 events per hour with OSA symptoms [8]. OSA syndrome is defined as OSA resulting in symptoms [9]. Symptoms of OSA include sleep related apnea symptoms such as snoring and choking and excessive daytime sleepiness due to the disturbed sleep [10,11]. The prevalence of OSA syndrome is 2–4%, while OSA occurs in up to 27% in middle aged men [10], the population at greatest risk for Barrett’s esophagus. Given the complexity and cost of overnight polysomnography, the Epworth sleepiness scale (ESS) is often used to assess daytime sleepiness and has been shown to correlate moderately with objective measures of sleepiness [12]. ESS consists of 8 items that ask how likely the respondent is to doze off or fall asleep in a variety of daytime situations [13]. The sleep apnea symptom index consists of 3 questions on the recent frequency of sleep-related apnea symptoms and performs well in the prediction of OSA [14]. OSA is associated with obesity, hypertension, cardiovascular disease, diabetes and gastroesophageal reflux [11,15,16]. Some studies have shown a decrease in gastroesophageal reflux symptoms after treatment of OSA with continuous positive airway pressure [17], although the results are conflicting [18,19]. It has been suggested that OSA increases reflux events by decreasing the esophageal pressure during OSA events [20–22]. The aim of this study was to test the hypothesis that OSA symptoms increase the risk of Barrett’s esophagus and that this association is mediated by gastroesophageal reflux or obesity.

## Methods

### Study participants

This study, entitled the Barrett’s Oesophagus Metabolic Study (BOMS), was nested within the Study of Digestive Health (SDH), a population-based study conducted in Brisbane, Australia between February 1, 2003 and June 30, 2006. These data collections have been described in detail elsewhere [5,23]. Briefly, SDH included cases aged 18–79 years with newly diagnosed Barrett’s esophagus (confirmed by histology as specialized intestinal metaplasia in a biopsy from the tubular esophagus). Controls were randomly selected from the same geographical regions from the Australian Electoral Roll (enrolment is compulsory by law in Australia) and matched for sex and age (in 5-year categories) of the cases with Barrett’s esophagus. Cases and controls were contacted by mail and asked if they would participate in BOMS, a study conducted in 2007–2009 which included a standardized interview by a research nurse, clinical measurements of height, weight, waist, hip, and neck circumference, and a self-completed written questionnaire. The questionnaire included questions regarding general health, tobacco smoking habits, sleep related apnea symptoms, and daytime sleepiness using the Epworth sleepiness scale [13]. Information regarding gastroesophageal reflux symptoms, medications and previous endoscopies were attained during the interview. Of all 359 patients with Barrett’s esophagus who participated in SDH, 69 (19%) declined to participate and 53 (15%) were found to be ineligible for BOMS, leaving 237 (66%) for the present study. Among the 419 originally identified controls, 108 (26%) declined to participate and 64 (15%) were found to be ineligible, leaving 247 (59%) for this study. Participants from SDH were ineligible for BOMS if they had developed esophageal adenocarcinoma since SDH, died, become too ill or mentally incompetent, had moved out of the study area, or were unable to be contacted.

### Approval by the Human Research Ethics Committee

The study was approved by the Human Research Ethics Committee of the Queensland Institute of Medical Research (The data collection BOMS on 15 January 2007 reference no P1304; SDH on 18 September 2002 reference no P514) and written informed consent was gathered from all participants.

### Assessment of Barrett’s esophagus

Barrett’s esophagus was defined as presence of intestinal metaplasia (columnar epithelium with goblet cells) in a biopsy taken from the esophagus during upper gastrointestinal endoscopy, regardless of the length of involved mucosa [24]. Only newly identified cases of Barrett’s were included in SDH.

### Assessment of symptoms of obstructive sleep apnea

Symptoms of OSA were measured in three ways. First, the Epworth sleepiness scale (ESS) was used to measure daytime sleepiness. The ESS consists of 8 items and the participants were asked how likely they were to doze off when they were in a variety of daytime situations. Each item had 4 response alternatives: “Would never doze”, “Slight chance of dozing”, “Moderate chance of dozing”, or “High chance of dozing”. The never doze alternative gave 0 points and the most frequent alternative gave 3 points. The scores were added together and participants with a total ESS score of ≥10 were defined as having excessive daytime sleepiness.

Secondly, the sleep apnea symptom index was used to assess sleep related symptoms of OSA. This index consists of 3 questions and the participants were asked how often during the last month they have had (or been told they have) the following sleep-related symptoms: “Snorting or gasping”, “Loud snoring”, or “Breathing stops, choke or struggle for breath”. For each item the participant could answer “Never”, “Rarely, less than once a week”, “1–2 times a week”, “3–4 times a week”, “5–7 times a week”, or “Don’t know”, with scores ranging from 0–4, where the alternative “Don’t know” was set as missing before the items were summarized and the mean values calculated. A mean score of <1 was defined as “never” having sleep apnea symptoms, 1-<2 as “rarely” having sleep apnea symptoms, and ≥2 as “often” having sleep apnea symptoms. This sleep apnea symptom index was created from a scale with 16 different sleep problem items [14].

Thirdly, we combined the categorized daytime sleepiness variable with the sleep-related symptoms of OSA in order to get a proxy for the stricter OSA *syndrome*. Participants were categorized into having both symptoms (present daytime sleepiness and frequent sleep-related apnea symptoms), one of the symptoms, or neither of the symptoms.

### Statistical analyses

Cases and controls were compared for sex, age (categorized in six groups <39, 40–49, 50–59, 60–69,70–79 and ≥80), body mass index (BMI; categorized according to the WHO classification as <25, 25–29.9 or ≥30.0 kg/m^2^), tobacco smoking (categorized as current, previous, or never smokers), education (categorized into high school, tech/trade college, or university), gastroesophageal reflux symptoms (worst ever symptoms categorized as “at least weekly symptoms” or “less than weekly symptoms”) and neck circumference (divided into sex-specific tertiles [t] defined by tertile cut-points in the distribution of controls). We used Pearson’s chi-square test to evaluate potential differences between cases and controls. For BMI and neck circumference, means and standard deviations were also calculated.

We then fitted separate multivariable logistic regression models to estimate the odds ratios (OR) and 95% confidence intervals (95% CI) for associations between excessive daytime sleepiness, sleep-related apnea symptoms, or the combination of these symptoms in relation to risk of Barrett’s esophagus. First, models adjusted for age and sex only were analyzed. We then constructed multivariable models further adjusting for BMI. Neck circumference were not added to the model due to high correlation with BMI (chi-square 249 p-value >.0001 as categories; Pearson’s correlation coefficient 0.56 p-value <.0001 when analyzed as continuous variables). In an additional model we further included gastroesophageal reflux symptoms (worst ever reflux symptoms categorized into “at least weekly symptoms” and “less than weekly symptoms”), as gastroesophageal reflux was assumed to be a mediator of a potential association between sleep disorders and Barrett’s esophagus. The analyses were performed using SAS version 9.2 (SAS institute, Cary, NC).

## Results

### Study participants

Characteristics of the 237 cases of Barrett’s esophagus and 247 controls are presented in Table 1. The distribution of age and sex were similar in the comparison groups due to the frequency matching. There was a higher proportion of obesity (BMI >30) among cases (35%) than among controls (23%), and measured neck circumference was higher in cases compared to controls. There was also a higher proportion of current cigarette smokers among the cases (14%) compared to controls (9%). Cases had a lower educational level than controls. The cases also had a higher proportion of at least weekly gastroesophageal reflux symptoms (85%) compared to controls (38%).

**Table 1:** Characteristics of cases of Barrett’s esophagus and population controls.

		Barrett’s esophagus	Controls	p-value^†^
Characteristics		n (%)	n (%)	
**All participants**		237 (100)	247 (100)	
**Sex**	Female	72 (30)	83 (34)	
	Male	165 (70)	164(66)	0.45
**Age (years)**	< 39	10 (4)	9 (4)	
	40–49	23 (8)	29 (12)	
	50–59	52 (22)	63 (26)	
	60–69	86 (36)	85 (34)	
	70–79	52 (22)	50 (20)	
	≥ 80	14 (6)	11(4)	0.85
**Body mass index**	< 25 kg/m^2^	41 (17)	68 (28)	
	25–29.99 kg/m^2^	114 (48)	122 (49)	
	≥ 30 kg/m^2^	82 (35)	57 (23)	0.004
	Mean (SD)	29 (5)	28 (5)	0.0202
**Neck circumference** ^**‡**^	Tertile 1	62 (26)	85 (34)	
	Tertile 2	71 (30)	81 (33)	
	Tertile 3	104 (44)	81 (33)	0.032
	Mean (SD)	39 (4)	39 (4)	0.0209
**Tobacco smoking**	Never smoker	79 (33)	142 (57)	
	Ex-smoker	124 (53)	84 (34)	
	Current smoker	34 (14)	21 (9)	<0.0001
**Education**	High school or lower	101 (43)	84 (35)	
	Tech/Trade College	103 (43)	96 (40)	
	University studies	33 (14)	63 (26)	0.004
**Gastroesophageal reflux symptoms** ^**§**^	< Weekly	36 (15)	152 (62)	
	≥ Weekly	199 (85)	93 (38)	<0.0001

^†^ Pearson’s chi-square test for categorized variables and student t-test for continuous variables.

^‡^ The cut-offs for neck circumference were tertiles (t): males t1 0–39.05 cm; t2 39.05–41.40 cm, t3 > 41.40 cm females t1 0–33.45 cm; t2 33.45–35.55 cm; t3 > 35.55 cm.

^§^The participants were asked if how often they had acid regurgitation or heartburn when the symptoms were most frequent.

### Excessive daytime sleepiness, sleep related apnea symptoms and risk of Barrett’s esophagus

The prevalence of excessive daytime sleepiness (24% vs. 18%; p-value 0.1142) and frequent sleep apnea symptoms (20% vs. 13%; p-value 0.1730) were higher among cases than controls. In the age- and sex-adjusted model, a non-statistically significantly risk of Barrett’s esophagus was seen for participants with excessive daytime sleepiness (OR 1.50, 95% CI 0.95–2.35) (Table 2). After adjustment for BMI, the OR was slightly attenuated (OR 1.42, 95% CI 0.90–2.34). When the model was also adjusted for gastroesophageal reflux symptoms, the OR was reduced to unity (Table 2). We observed similar patterns with the measure of sleep apnea symptoms; there was a possible association in the age- and sex-adjusted model that did not reach statistical significance (OR 1.59, 95% CI 0.91–2.78), and the OR was somewhat attenuated by adjusting for BMI, and further lowered after adjusting for gastroesophageal reflux symptoms (Table 3).

**Table 2:** Excessive daytime sleepiness and the risk of Barrett’s esophagus measured with odds ratio (OR) and 95% confidence interval (CI).

		Barrett’s esophagus cases	Controls	Minimally adjusted^†^	Partially adjusted^‡^	Fully Adjusted^§^
		n (%)	n (%)	OR (95% CI)	OR (95% CI)	OR (95% CI)
**Excessive daytime sleepiness** ^¶^	Absent	181 (76)	203 (82)	1 (reference)	1 (reference)	1 (reference)
	Present	56 (24)	44 (18)	1.50 (0.95–2.35)	1.42 (0.90–2.34)	1.02 (0.61–1.70)

^†^Adjusted for age and sex

^‡^Adjusted for age, sex and body mass index

^§^Adjusted for age, sex, body mass index and gastroesophageal reflux symptoms

^¶^Measured with the Epworth sleepiness scale (categorized <10 absent, ≥ 10 present).

**Table 3:** Sleep related apnea symptoms and the risk of Barrett’s esophagus measured with odds ratio (OR) and 95% confidence interval (CI).

		Barrett’s esophagus cases	Controls	Minimally adjusted^†^	Partially adjusted^‡^	Fully Adjusted^§^
		n (%)	n (%)	OR (95% CI)	OR (95% CI)	OR (95% CI)
**Sleep related apnea symptoms**	Never	107 (55)	125 (58)	1 (reference)	1 (reference)	1 (reference)
	Rarely	50 (26)	61 (29)	0.95 (0.60–1.50)	0.87 (0.54–1.38)	0.76 (0.44–1.31)
	Often	39 (20)	28 (13)	1.59 (0.91–2.78)	1.32 (0.74–2.36)	0.72 (0.38–1.38)

^†^Adjusted for age and sex

^‡^Adjusted for age, sex and body mass index

^§^Adjusted for age, sex, body mass index and gastroesophageal reflux symptoms.

### The combination of excessive daytime sleepiness, sleep related apnea symptoms and risk of Barrett’s esophagus

When a combination of excessive daytime sleepiness and sleep-related apnea symptoms was analyzed, the age and sex adjusted model showed a more than doubled increased risk of Barrett’s esophagus (OR 2.35, 95% CI 0.94–5.85), although not statistically significant (Table 4). The OR was slightly attenuated when BMI was added to the model (OR 2.02, 95% CI 0.80–5.11) and vanished when gastroesophageal reflux was added (OR 0.81, 95% CI 0.31–2.15) (Table 4).

**Table 4:** A combination of sleep related apnea symptoms and excessive daytime sleepiness and the risk of Barrett’s esophagus measured with odds ratio (OR) and 95% confidence interval (CI).

		Barrett’s esophagus cases	Controls	Minimally adjusted^†^	Partially adjusted^‡^	Fully Adjusted^§^
		n (%)	n (%)	OR (95% CI)	OR (95% CI)	OR (95% CI)
**Combination Sleep related apnea symptoms andDaytime sleepiness**	None	88 (45)	106 (50)	1 (reference)	1 (reference)	1 (reference)
	Either symptom	93 (47)	100 (47)	1.12 (0.75–1.67)	1.00 (0.66–1.52)	0.78 (0.48–1.27)
	Both symptoms	15 (8)	8 (4)	2.35 (0.94–5.85)	2.02 (0.80–5.11)	0.81 (0.31–2.15)

^†^Adjusted for age and sex

^‡^Adjusted for age, sex and body mass index

^§^Adjusted for age, sex, body mass index and gastroesophageal reflux symptoms.

## Discussion

This study indicates that excessive daytime sleepiness, sleep-related apnea symptoms and a combination of symptoms are more common among patients with Barrett’s esophagus than population controls, although no statistically significant associations were found. The risk estimates were slightly attenuated by adjustment for BMI, but were abolished after adjustment for gastroesophageal reflux symptoms. This suggests that the association between OSA symptoms and Barrett’s esophagus is mediated by gastroesophageal reflux.

Strengths of the study include a strict case assessment of Barrett’s esophagus with clear endoscopic and histological criteria, recruitment of cases and controls from the same geographical area, and an objective assessment of anthropometric measures. There are also several limitations. The cases were diagnosed with Barrett’s esophagus at recruitment, which was up to 7 years before assessment of the study exposures sleep apnea and daytime sleepiness, as well as the potential confounding factors BMI and tobacco smoking. Because participation in the original study and this substudy was less than complete, there is the potential that selection bias may have introduced spurious associations where none exist in reality. In the absence of data on non-participants, it is impossible to know in which direction such a bias might operate. There is also a potential risk of bias due to lifestyle changes after diagnoses, e.g. reduction in smoking and changes in eating habits, which may have influenced obesity and sleep patterns. Test of the correlation between measure of lifestyle factors at time of recruitment (2003–2006) and measures included in this study (2007–2009), however, have shown good correlation for both cases and controls [5]. Another limitation was the lack of polysomnographic data for an objective measure of OSA, but we used the subjective Epworth sleepiness scale (ESS) and self-reported sleep related apnea symptoms as markers of those at an increased risk of OSA. The ESS correlates weakly with objective measures of obstructive sleep apnea severity such as the apnea hypopnea index or oxygen saturation but moderately with objective measures of daytime sleepiness [12]. The self-reported sleep apnea symptom index has been shown to predict OSA [14,25]. Even so, the index relies on self-reported information; future studies might consider validating self-reports by obtaining additional information from a partner. Finally, there was limited statistical power to ascertain weak or moderately strong associations.

Few studies have explored the potential association between sleep apnea and Barrett’s esophagus. In a recent US study, including 73 Barrett’s cases and 242 controls (66 upper endoscopy controls and 171 colonoscopy controls), a 2-fold increased risk of OSA was found for Barrett’s esophagus (OR 2.08, 95% CI 1.12–3.88), but the association was attenuated and statistically non-significant after adjustment for age and BMI (OR 1.51, 95% CI 0.72–3.15) [26]. In another US study, including 7,590 patients who underwent polysomnography and upper endoscopy during a 12-year period, a moderately increased risk of Barrett’s esophagus was found among OSA patients (OR 1.4, 95% CI 1.1–1.7) [27], but no adjustment for BMI was conducted. In a third US study including 7,482 participants who had also had polysomnography and upper endoscopy showed an 80% increased occurrence of Barrett´s esophagus if they had OSA (OR 1.8; 95% CI 1.1–3.2), after adjustment for sex, age, BMI, smoking and gastroesophageal reflux [28]. The increased point estimates in our study were abolished after adjustment for gastroesophageal reflux symptoms, suggesting that the mechanism behind a possible association is related to gastroesophageal reflux.

It is well known that gastroesophageal reflux is the main causal factor of Barrett’s esophagus and esophageal adenocarcinoma [6,7,29], and gastroesophageal reflux has been associated with sleep disturbance, insomnia [30], and OSA [15,17,19], although there are some conflicting results for OSA [18,31]. In a small case-control study, OSA patients had more nocturnal gastroesophageal reflux events and longer esophageal exposure to esophageal acidity (pH <4) [15]. Treatment with continuous positive airway pressure (CPAP), the standard treatment for OSA, reduced gastroesophageal reflux symptoms in OSA patients [17,19], which might indicate a causal link. However, it has also been shown that CPAP reduces gastroesophageal reflux regardless of OSA-status [15], which contradicts such interpretation.

It has been suggested that decreased intra-esophageal pressure during OSA events creates a difference in pressure between the esophagus and the stomach, thereby promoting episodes of gastroesophageal reflux, but laboratory studies have shown that compensatory changes occur in the upper esophageal sphincter and the gastroesophageal junction which prevent pressure differences [21], and that nocturnal gastroesophageal reflux events are instead caused by transient lower esophageal sphincter relaxations [21].

In conclusion, it remains possible that symptoms of OSA are moderately associated with risk of Barrett’s esophagus, although this association is likely to be mediated by gastroesophageal reflux. Additional studies, including analyses of pooled data from existing datasets, would assist in clarifying the complex correlations that underpin these clinical observations.
